# Nano-Priming for Inducing Salinity Tolerance, Disease Resistance, Yield Attributes, and Alleviating Heavy Metal Toxicity in Plants

**DOI:** 10.3390/plants13030446

**Published:** 2024-02-03

**Authors:** Jisun H. J. Lee, Deepak M. Kasote

**Affiliations:** 1 Department of Plant Science and Technology, Chung-Ang University, Anseong 17546, Republic of Korea; 2 Agricultural Research Station, Qatar University, Doha P.O. Box 2713, Qatar

**Keywords:** nano-priming, salinity stress, disease resistance, heavy metal toxicity, phytotoxicity, environment safety

## Abstract

In today’s time, agricultural productivity is severely affected by climate change and increasing pollution. Hence, several biotechnological approaches, including genetic and non-genetic strategies, have been developed and adapted to increase agricultural productivity. One of them is nano-priming, i.e., seed priming with nanomaterials. Thus far, nano-priming methods have been successfully used to mount desired physiological responses and productivity attributes in crops. In this review, the literature about the utility of nano-priming methods for increasing seed vigor, germination, photosynthetic output, biomass, early growth, and crop yield has been summarized. Moreover, the available knowledge about the use of nano-priming methods in modulating plant antioxidant defenses and hormonal networks, inducing salinity tolerance and disease resistance, as well as alleviating heavy metal toxicity in plants, is reviewed. The significance of nano-priming methods in the context of phytotoxicity and environmental safety has also been discussed. For future perspectives, knowledge gaps in the present literature are highlighted, and the need for optimization and validation of nano-priming methods and their plant physiological outcomes, from lab to field, is emphasized.

## 1. Introduction

Climate change and increasing pollution are exerting significant pressure on contemporary agriculture, impacting its output. Biotic risks, such as agricultural pests, are another determinant of agricultural productivity, which is also parallelly increased with climate change’s measurable factors, mainly rising temperature, changing precipitation patterns, and atmospheric CO_2_ levels [1]. It has been estimated that the total global food demand will increase by 35–56% between 2010 and 2050, and existing crop production will need to expand by up to 60% by 2050 to ensure food security [2,3]. Along with food production, providing nutritional security is another big challenge facing the current agricultural setup. In short, present and future agriculture will have several challenges to overcome to be sustainable. So far, several strategies, including both genetic and agronomic methods, have been developed and adapted to increase the overall agricultural output both qualitatively and quantitively. Amongst these, the use of nanotechnology in agriculture is found to be a revolutionary strategy to cope with increasing agricultural productivity in the background of biotic and abiotic risk factors.

In agriculture, nanotechnology has been used for various purposes [4]. The initial application of nanotechnology in the agricultural sector was related to the development of biosensors, to detect the quality of agricultural products and pesticide residues [5]. These days, synthetic nanomaterials are used as agents to protect plants from pathogens and to stimulate plant growth. In addition, nano-systems, such as hydrogels, nano-clays, and nano-zeolites, have been successfully used to improve the quality of the soil [6]. Similarly, nanoscale systems are supported on different inorganic adsorbents and metallic nanoparticles (NPs) are also used to remove/degrade soil contaminants through redox reactions [7]. The nanoformulations of pesticides like avermectin, bifenthrin, and validamycin are found to be effective in agriculture [8,9]. Despite several positive applications, the large-scale use of nanomaterials in agriculture has stimulated many reservations. This is mainly because little is known about the environmental and agricultural toxicity of nanomaterials, including their impact on food safety. Considering this, the controlled and minimal use of nanomaterials could be promising in agriculture.

Because of easiness and effectiveness, the technique of seed priming with nanomaterials, typically called nano-priming, has been gaining importance in agriculture. Thus far, nano-priming techniques have been used to improve seed germination, plant disease resistance, stress tolerance, and yield [10,11]. Moreover, this technique is highly controllable, compared to the foliar application of nanomaterials. Hence, nano-priming can be considered more environmentally friendly and less phytotoxic. The safety of nano-priming methods can be further improved using green synthetic nanomaterials, as they may minimize environmental risks and the human toxicity concerns that arise for nanoparticles [12].

In this article, the recent literature on the utility of various seed nano-priming methods, including the use of nanomaterials for increasing seed vigor, germination, photosynthetic output, biomass, early growth, and yield, has been summarized. Also, research works on the use of nano-priming methods in modulating plant antioxidant defense and hormonal networks, inducing salinity tolerance and disease resistance, as well as alleviating heavy metal toxicity in plants have been reviewed. Moreover, the significance of nano-priming techniques in minimizing environmental and agricultural toxicity has also been discussed.

## 2. Nanomaterials Used in Seed Priming

Seed priming (presoaking) is an age-old technique used to enhance germination, seedling development, and crop yield under normal and adverse conditions [13]. Various seed priming techniques, such as hydro-priming, halo-priming, osmo-priming, bio-priming, solid matrix priming, chemo priming, thermopriming, hormonal priming, and nano-priming, have been developed, and are in practice as well [14]. Amongst these, nano-priming is a novel and more effective technology [15]. Recent studies have shown the utility of nano-priming in enhancing seed germination, growth, and yield, including modulating physiological responses such as antioxidant systems and hormonal pathways in seeds and plants [16]. Nano-priming methods are found to influence plant physiology at cellular, organ, and plant levels. So far, various nanomaterials of biogenic and synthetic metallic compounds and chitosan NPs have been used as seed-priming agents [12,17]. Among these, inorganic nanomaterials (metal and metal oxides), mostly metal oxides, have been preferably used as priming agents in nano-priming methods. This might be because metal oxide NPs tend to exhibit higher solubility than their metallic counterparts. Consequently, they are more readily available and can be easily translocated within plants [18]. Table 1 summarizes seed priming studies using various nanomaterials and their effects on plant physiological responses and productivity. These studies indicated that the physiological outcome of each nano-priming method is plant genotype-specific, and mainly linked with the composition, size, shape, and applied concentration of nanomaterials. 

## 3. Nano-Priming to Induce Yield Attributes

### 3.1. Nano-Priming to Boost Seed Vigor, Germination, and Early Growth

Seed vigor is a complex trait that defines how quickly seeds germinate in the field under different environmental conditions [44]. This includes seed longevity, germination speed, seedling growth, and early stress tolerance [45]. Hence, seed vigor is a crucial parameter, which has a profound impact on sustainable and profitable agriculture [46]. Recent studies showed that various nano-priming treatments enhanced seed vigor, germination, and early growth in both lab and field conditions, summarized in Table 1. The impact of nano-priming treatments on seed vigor, germination, and early growth was found to be dependent on the priming concentration and can be enhanced with the co-addition of other compounds. In corn, the observed effect of seed priming with trimanganese tetraoxide (Mn_3_O_4_)-NPs on seed vigor and germination was concentration-dependent and even more positive compared to its bulk counterparts. The highest effect on germination rate, vigor, dry biomass, and length was observed at 20 mg L^−1^ [19]. In a field experiment, the combinations of zinc oxide (ZnO)-NPs with sodium selenite and sodium selenate showed synergistic action and improved seed vigor, metabolic profiles, nutrient uptake, growth, and yield of direct seeded rice [22].

**Figure 1:**
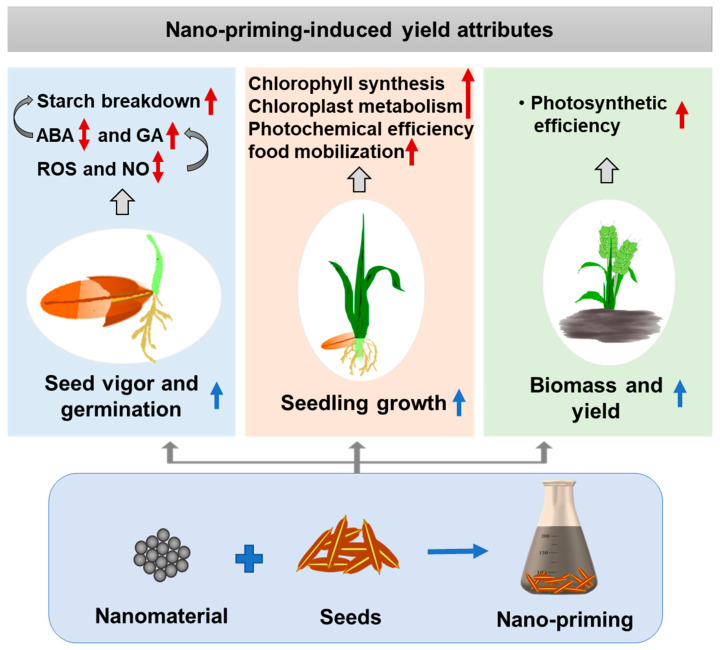
Pictorial representation of the influence of nano-priming methods on increasing yield attributes in crops. Nano-priming methods may induce seed vigor and germination by increasing the optimal production of reactive oxygen species (ROS) and nitric oxide (NO), thereby enhancing the production of gibberellic acid (GA), including modulating biosynthesis of abscisic acid (ABA). This collectively increased the starch breakdown in primed seeds. Enhanced seedling growth after nano-priming treatments may be associated with increased chlorophyll synthesis, chloroplast biosynthesis, and photochemical efficiency, including food mobilization. Nano-priming methods may increase the biomass and yield of crops by enhancing photosynthesis. Up arrows denote increased levels, and the up-down arrow indicates modulated levels.

Plant hormones and free radicals play crucial roles in seed vigor and germination, including early growth. The plant hormone abscisic acid (ABA) is involved in the early stages of germination, and gibberellic acid (GA) plays a valuable role in the late stages of germination [47]. The significance of free radicals, such as reactive oxygen species (ROS) and reactive nitrogen species (RNS) is also well-established in seed germination [47]. In seeds, ROS play several functions such as endosperm weakening, seed reserves mobilization, and providing protection from pathogens, and also act as a messenger of environmental cues during seed germination [48]. In rapeseed, seed priming with selenium (Se) and ZnO NPs enhanced germination attributes immediately after sowing by modulating the expression of ABA (BnCYP707A1, 3, and 4) and GA (*GA20ox* and *GA3ox*) genes [36]. Priming rice seeds with nanoscale zerovalent iron (nZVI) at a concentration of 20 mg L^−1^ for 72 h modulated the production of intracellular ROS to enhance seed vigor and germination [49]. In seeds, nZVI could generate higher levels of ROS via Fenton’s reaction, and because of higher ROS, nitrate reductase activity was increased, and, thereby, the nitric oxide (NO) level. nZVI-primed sets, elevated optimal levels of ROS, and NO significantly up-regulated genes such as OsGA3Ox2 and OsGAMYB, which are responsible for GA biosynthesis and GA-induced signaling. Because of this, the activity of hydrolytic enzymes were significantly increased, leading to the enhanced mobilization of nutrients and rate of seed germination [49]. However, the authors suggested that further research is needed to understand the relationship between ROS perception and signaling molecules involved in nano-priming-mediated seed germination [49]. In another study, seed priming with iron oxide (FeO) NPs was shown to increase starch breakdown during seed germination and growth [23]. Taken together, available literature suggests that starch metabolism, ROS production, and the biosynthesis of ABA and GA hormones can be the key molecular targets for primed nanomaterials to modulate seed vigor, germination, and early growth (Figure 1). However, the overall existing information in this regard is very limited. Further detailed studies are warranted on the optimization of nano-priming methods (doses and priming times) and how nanomaterials having various properties affect these biochemical events in the seeds of different plant genotypes.

### 3.2. Nano-Priming to Induce Photosynthetic Output, Biomass, and Yield

Increasing growth and photosynthetic rates are the key processes to enhance the biomass and yield of plants [50]. Seed priming with metallic NPs, such as iron (Fe), manganese (Mn), zinc (Zn), and silicon (Si), has been found to modulate chlorophyll synthesis and photosynthetic apparatus in plants [12,15,42]. In maize and watermelon, the positive effect of seed priming with ZnO and manganese oxide (MnO) NPs on photosynthesis was associated with increasing chlorophyll contents and photochemical efficiency [15,42]. It has been hypothesized that ZnO NPs modulated chlorophyll synthesis by being involved in protochlorophyllide formation [51]. Seed priming with Fe NPs promoted chlorophyll synthesis and chloroplast metabolism and thereby improved carbon assimilation [27]. At optimal doses, Fe_2_O_3_ NPs increased the conversion efficiency of primary light energy of PS II (Fv/Fm) and water use [27]. However, the application of higher nano-priming concentrations of ferric oxide (Fe_2_O_3_) NPs was found to disrupt chloroplast structure and reduce photosynthesis in *Kobresia capillifolia* (Decne.) C.B.Clarke, including a decrease in starch accumulation [27]. Altogether, the positive or negative impact of metallic NPs on chlorophyll synthesis, including overall photosynthesis, was found to be dependent on their type, concentration used, and plant genotypes [12,15]. Moreover, considering our limited understanding, further detailed studies are needed about the molecular mechanism by which these beneficial metallic NPs influence the photosynthetic apparatus of plants.

Photosynthetic efficiency positively correlated with biomass production in plants. Also, the role of plant nutrients, such as Fe and Mn, in modulating photosynthetic apparatus was studied [52]. Hence, the impact of seed priming with nutrient NPs on biomass production was also studied. Dry biomass was found to be increased after seed priming with Mn_3_O_4_ and ZnO NPs in corn and maize, respectively [19,42]. Likewise, nano-priming was also found to increase crop yield in field trials. Onion seeds primed with gold (Au) NPs were found to increase the average yield by 23.9%, compared to unprimed seeds [24]. The common molecular processes modulated by nano-priming methods while enhancing seedling growth, biomass, and yield are collectively depicted in Figure 1.

## 4. Nano-Priming to Mount Physiological Responses

### 4.1. Nano-Priming to Modulate Hormonal Responses

Phytohormones have a crucial role in regulating plant development and providing tolerance or susceptibility against abiotic and biotic stresses [53]. Plant hormones, such as ABA, salicylic acid (SA), GA, and jasmonates (JAs) were found to regulate abiotic stress responses [54]. Hence, modulating the plant hormone network can provide a unique window of opportunity for crop improvement. Studies have shown the utility of nano-priming methods in modulating plant hormone networks. In the previous section, we have seen that seed priming with SeO, ZnO and FeO NPs influenced the biosynthesis of ABA and GA hormones and modulated seed vigor and germination [36,49]. Seed germination decision was found to be dominated by the balance of competing ABA and GA hormone signaling pathways [54]. Moreover, both ABA and GA are also considered key hormones for the regulation of plant responses to environmental signals. ABA is considered to be a main plant hormone that cross-talks with GA, auxin, cytokinins, brassinosteroids, and jasmonic acid (JA), and regulates plant adaptability in drought and salt stresses [55]. JA and its precursors and derivatives, collectively called Jas, are a group of plant hormones that play a vital role in plant response to abiotic and biotic stresses [56]. JA crosstalk with various other plant hormones, such as ABA, GA, and SA, regulates the balance between plant growth and defense [56]. In watermelon seedlings, seed priming with FeO NPs was found to be useful in inducing jasmonates-linked defense responses. The FeO NP priming treatments considerably modulated the level of 12-oxo phytodienoic acid (OPDA) in diploid and triploid watermelon seedlings [12]. However, these treatments had no impact on the levels of ABA, GA, JA, and zeatin hormones [12]. OPDA is a precursor of JA that accumulates under salt stress conditions and also helps to promote seed dormancy [57]. JA, with various other plant hormones, regulates the balance between plant growth and defense. Similarly, rapeseed priming with polyacrylic acid-coated cerium oxide (CeO_2_) NPs elevated salicylic acid (SA) contents in the roots and shoots by upregulating the expression of SA biosynthesis-related genes under salt stress [28]. However, it is unclear how CeO_2_ NPs modulate SA, and this modulated SA brings physio-biochemical and molecular changes under salt stress. Considering the overall limited available knowledge in this context, investigating the molecular mechanisms by which various nano-priming methods modulate hormonal responses, including hormonal cross-talk, especially under stress conditions, will be an interesting theme of research.

### 4.2. Nano-Priming to Modulate Plant Antioxidant Defense

**Figure 2:**
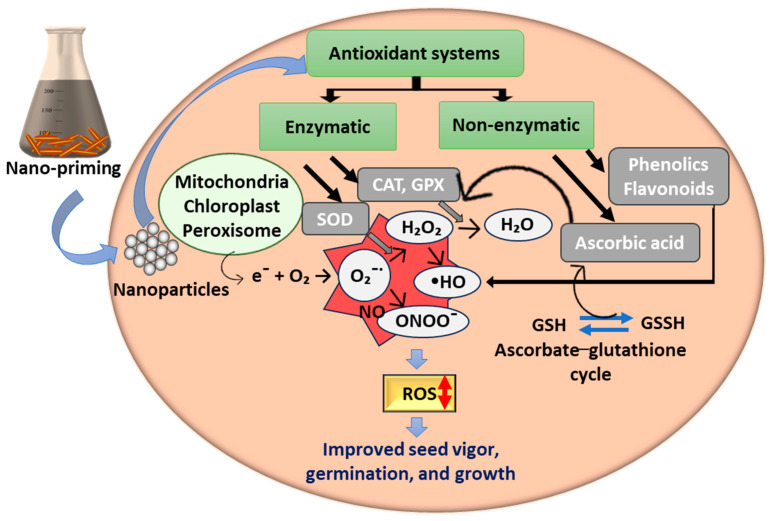
Molecular mechanisms involved in nano-priming methods-based modulation of antioxidant systems in response to the regulation of reactive oxygen species (ROS). Nanoparticles used in seed priming methods modulated ROS (superoxide anion (O_2_^−^˙), hydroxyl radical (•OH), hydrogen peroxide (H_2_O_2_), and peroxynitrite (ONOO^−^)) levels, using both enzymatic and non-enzymatic antioxidant systems, and improved seed vigor, germination, and initial seedling growth.

Plants have effective antioxidant defense systems (enzymatic and non-enzymatic) to overcome the toxic effects of overproduced free radicals, such as ROS [58]. Enzymatic systems include enzymes like superoxide dismutase (SOD), catalase (CAT), peroxidase (POD) glutathione peroxidase (GPx), and glutathione reductase (GR). In contrast, non-enzymatic systems consist of low molecular weight antioxidants (ascorbic acid, proline, phenolic acids, and flavonoids) and high molecular weight secondary metabolites such as tannin [58]. Under normal conditions, plant cells produce an optimal amount of ROS as signaling molecules. ROS play a crucial role in abiotic and biotic stress-related events and are also involved in numerous processes of seed germination and plant development [59]. The nano-priming methods have been effectively used to halt the overproduction of ROS in plant cells, including optimizing intracellular ROS generation. In seeds, optimal ROS generation supports early seed germination by acting as messengers in the cell signaling processes that weaken endosperm and mobilize stored food [49]. Rice seeds primed with nano-scale zerovalent iron (G-nZVI) (20 mg L^−1^, for 72 h) regulated intracellular ROS and NO levels, and promoted seed vigor and germination rate by orchestrating all metabolic activity [49]. On e contrary, antioxidant enzyme activity (SOD, CAT, and POD) were also found to be increased in the young seeds to maintain ROS homeostasis, which contributed to the enhanced seed vigor. Similarly, increased activity of antioxidant enzymes in rice seedlings and wheat grains was also observed when they were primed with silver (Ag) and Si NPs, respectively [40,43]. Along with these, the enzymes of the ascorbate–glutathione (AsA–GSH) cycle play a crucial role in protecting the cells from oxidative stress [40]. These increased activities of antioxidant enzymes lead to reduced levels of malondialdehyde (MDA), an indicator of lipid peroxide, electrolyte leakage (EL), and hydrogen peroxide (H_2_O_2_), including ROS. In watermelon seedlings, an increase in non-enzymatic antioxidant profiles was also found at higher doses of MnO NP priming [15]. The phyto-toxicity of nanomaterials has been linked with elevated levels of non-enzymatic antioxidants, such as ascorbic acid, total phenolics, and flavonoids [40]. Seed genotype and priming concentration are crucial factors that define the positive or negative influence of nano-priming methods on antioxidant systems [15]. Along with this, the size of NPs also determines the influence of nano-priming on the plant antioxidant systems. In primed rice seedlings, Ag NP-size-dependent enhancement in the activity of antioxidant enzymes (SOD, CAT, ascorbate peroxidase (APX), glutathione peroxidase, and glutathione reductase) was observed. Among the different sizes studied (20–150 nm), larger size Ag NPs (150 nm diameter) induced the increased activity of antioxidant enzymes [31]. Taken together, the available literature suggests that the beneficial effects of the nano-priming methods are linked with their ROS-modulating potential through the enhancement of the enzymatic antioxidant system. Non-enzymatic antioxidants also regulate ROS levels directly, or mostly in conjunction with the primary antioxidant defense (enzymatic). This could be the reason that their levels significantly increased when all antioxidant defense collapsed at a time of toxicity. However, molecular mechanisms by which both enzymatic and non-enzymatic antioxidant systems regulate ROS levels in response to various types of NPs is not yet clear. The pictorial representation of molecular events involved in the nano-priming-based modulation of antioxidant defenses and, thereby, the regulation of ROS levels is shown in Figure 2.

### 4.3. Nano-Priming to Develop a Tolerance against Salinity Stress

**Figure 3:**
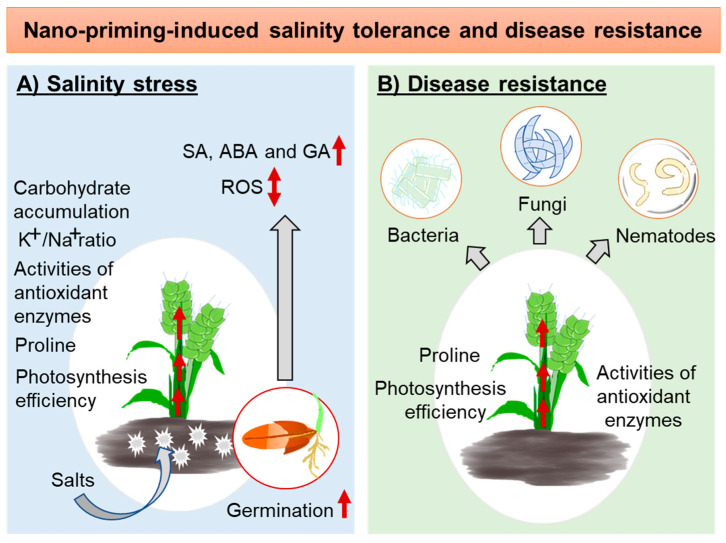
Schematic representation of molecular events induced or modulated by nano-priming methods during rendering, regarding salinity tolerance and disease resistance in plants. (**A**) Nano-priming methods may alleviate salinity stress during germination by modulating ROS production and the synthesis of abscisic acid (ABA), salicylic acid (SA), and gibberellic acid (GA) hormones. Similarly, nano-priming methods may induce salinity tolerance in plants by increasing photosynthesis, proline content, activity of antioxidant enzymes, K^+^/Na^+^ ratio, and carbohydrate accumulation. (**B**) Nano-priming methods can provide disease resistance against bacteria, fungi, and nematodes in plants by enhancing photosynthesis efficiency, proline content, and the activity of antioxidant enzymes. Up “red” arrows denote increased levels, and the up-down “red” arrow indicates modulated levels.

Drought, salinity, and extreme temperature are the main abiotic stresses that considerably influence crop productivity. Among these abiotic stresses, salinity causes a profound impact on seed germination and seedling growth [60]. Seed priming is found to be a useful method to decrease the negative effects of salinity [38]. Consequently, several seed priming techniques, including nano-priming, have been developed to cope with salinity stress. The cellular balance between sodium and potassium is crucial for plants in saline soil [61]. Under salinity stress, the plant experiences hyperosmotic shock and ionic imbalance that leads to oxidative stress [62]. Seed priming techniques are used to mount different defense mechanisms, such as an antioxidant defense and osmotic adjustment against salinity [60]. In wheat seedlings, seed priming with copper–chitosan (Cu–C) NPs mitigated polyethylene glycol-induced hyperosmotic stress and salinity by controlling the expression of enzymatic antioxidants and suppressing malondialdehyde (MDA) [35]. Similarly, seed priming with Se and ZnO NPs protected rapeseed cultivars from salt stress by increasing the activity of antioxidant enzymes. In wheat and *Lathyrus odoratus* L., nano-primed seedlings mitigated salinity stress by improving growth traits, K^+^/Na^+^ ratio, carbohydrate accumulation, enhanced photosynthetic pigments, and nonenzymatic antioxidant levels [35,63]. The effects of nano-priming on phytohormones, mainly GA and ABA, during seed germination have also been studied, since these hormones are known to have a significant effect on seed germination [64]. Rapeseed primed with Se and ZnO NPs provided salinity tolerance by modulating the expression levels of ABA and GA genes and improving the expression of genes (*BnCAM*, *BnPER*, *BnEXP4*, and *BnRAB28*) related to seed germination. However, the ways in which different nano-priming methods modulated different genes associated with salt tolerance deserve to be further explored. Moreover, these treatments are also found to modulate ROS production during the imbibition and germination stages, including improving cell wall loosening and cation toxicity tolerance [36]. ABA is the main plant hormone in salt and osmotic response. Nevertheless, how it interacts with other phytohormone signaling pathways in guarding cells and root tissues, including regulating growth, is unclear [61]. In pearl millet, Ag NP nano-priming significantly reduced salinity stress by increasing relative water and proline contents [34]. An increase in proline content was also observed in milk thistle seedlings upon chitosan nano-priming [38]. Proline, as a compatible solute, plays an important role in providing salt tolerance to the plant. However, the role of proline in salinity tolerance is debatable [65]. The summary of molecular events influenced by nano-priming methods and thereby providing salinity tolerance to plants are depicted in Figure 3A.

### 4.4. Nano-Priming to Mount Disease Resistance

Annual crop loss due to pathogen infestation is substantially high (20–40%), which involves pathogenic organisms, such as fungi, viruses, bacteria, nematodes, and oomycetes [66,67]. Several chemical and biological control strategies, including the use of disease-resistant crops, have been recommended to manage crop diseases. Recently, as an alternative strategy, nanotechnology-based crop disease management methods have also received attention. Seed priming with various nanomaterials has been used to mount disease resistance against fungal, bacterial, and nematode pathogens in crops (Figure 3B) [31,33,34]. In cumin, seed priming with chitosan and Ag NPs enhanced the activity of antioxidant enzymes such as SOD, CAT, guaiacol peroxidase, and polyphenol oxidase, and thereby restrained Fusarium wilt infection [68]. In another study, tomato seeds primed with Se NPs exhibited significant protection (72.9%) against late blight disease through the accumulation of lignin, callose, and H_2_O_2_, which serve as the cellular defense [33]. In the same study, elevated levels of SOD, lipoxygenase (LOX), β-1,3-glucanase (GLU), and phenylalanine lyase (PAL) were also reported, which were responsible for the acquired biochemical defense in the primed plants [33]. The inoculation of tomato with bacterial and fungal pathogens such as bacteria *Xanthomonas campestris* pv. *vesicatoria*, and *Pseudomonas syringae* pv. tomato, and fungi *Alternaria solani* and *Fusarium oxysporum* f.sp. lycopersici reduced plant growth, carotenoid and chlorophyll contents, and increased proline and the activity of SOD, PAL, CAT, and APX. Seed priming with titanium dioxide (TiO_2_) NPs (0.20 mL L^−1^) was found to improve these infection-afflicted plants’ growth, including levels of carotenoid, chlorophyll, proline, SOD, CAT, APX, and PAL, and also reduced disease indices [69]. Under greenhouse conditions, it was found that seed priming with silicon dioxide (SiO_2_) NPs (200 mg L^−1^) helped to reduce *Pectobacterium betavasculorum*, *Meloidogyne incognita*, and *Rhizoctonia solani* disease complex of beetroot. Seed priming with SiO_2_ NPs enhanced growth, photosynthetic performance, and the activity of CAT, SOD, PAL, and PPO, thereby reducing galling, nematode multiplication, and disease indices [34]. In field experiments, seed priming with novel synthesized 1,2,4-triazolyldithiocarbamate-conjugated Ag NPs reduced the incidence of bakanae disease in rice [32]. Findings about the effectiveness of seed priming with nanomaterials over their foliar spray are contradictory [34,69].

## 5. Nano-Priming to Alleviate the Heavy Metals Associated with Soil Pollution

Climate change and anthropogenic activities are severely affecting agricultural output. Soil pollution due to heavy metals contamination is the leading cause of crop yield loss. Heavy metals, such as cadmium (Cd), cobalt (Co), lead, chromium, and so on, cause phytotoxicity upon their accumulation in the soil. Among various heavy metal toxicity mitigation strategies, the nano-priming technique has been found to be promising. Seed priming with ZnO NPs considerably reduced Co stress and Cd toxicity in maize and rice, respectively [41,42]. The observed protective effect of ZnO NPs against Co stress was attributed to decreasing the uptake of Co and providing stability to plant ultra-cellular structures and photosynthetic apparatus [42]. Cd showed negative effects on seed germination and seedling growth in rice. The toxicity of Cd has been linked to the enhanced production of ROS and immobilization of starch, altering physiology and protein profiles [41,70]. Seed priming with ZnO NPs mitigated the Cd toxicity in rice seedlings by activating amylase, increasing the levels of antioxidant enzymes, promoting metallothionein formation, and reducing the MDA accumulation in the plant [41]. It is postulated that Zn competes with Cd and reduces its toxic level in the seed. Like ZnO NPs, seed priming with Si NPs reduces the Cd toxicity in wheat by reducing ROS levels and Cd concentration in grains [43]. Similar to Zn, Si competes with Cd and reduces its uptake and translocation in wheat [43]. Taken together, nano-priming can be a potential method to alleviate the heavy metals associated with soil pollution. However, further detailed studies focusing on the molecular mechanisms responsible for these positive effects in different plant genotypes are essential.

## 6. Nano-Priming for Micronutrients Biofortification

Malnutrition, particularly undernutrition, is the leading cause of poor health and death in the Global South. From a broader perspective, biofortification, which simply means increasing the density of nutrients in a crop through plant breeding, transgenic techniques, or agronomic practices, is considered an efficient means to deal with malnutrition. Agronomic biofortification may be beneficial over genetic biofortification, because genetic biofortification is a time-consuming method and there is limited available diversity in the targeted crop gene pool for the adaptation of this technology [71]. Among agronomic approaches, a technique of using engineered nanomaterials as seed treatments to biofortify crops is receiving considerable attention [72]. Ag NP nano-priming was found to be useful in increasing essential amino acid contents in cabbage leaves. In the same study, an increase in Fe content (23.8%) was also reported in cabbage leaves [26]. Significant increases in the grain Fe and zinc (Zn) contents were reported in wheat and maize, respectively, upon nano-priming with Fe_2_O_3_ and ZnO NPs [21,39]. Interestingly, the accumulation of Fe in wheat grains has been reported in both low- and high-Fe genotypes [39]. Nano-priming can be a potential technique that is quick and easy for the selective biofortification of micronutrients. However, only limited literature is available in this regard, and studies related to the translocation, distribution, and loading of micronutrients in the edible portion of plants, including their safety, will be of interest in the future.

## 7. Nano-Priming in the Context of Phytotoxicity and Environment Safety

Undoubtedly, nano-priming can be positively used to mount desired physiological and productivity attributes in crops. However, on the contrary, some studies also showed negative effects, mainly phytotoxicity, on some of the nano-priming methods [15,21]. Most of the observed phytotoxic or negative effects of nano-priming methods were dependent on their chemical composition, applied concentration, and size, and the degree of effects varied from genotype to genotype [15,21,31]. This fact indicates that the negative effects of nano-priming methods can be manageable by choosing proper nanomaterials and their application concentrations.

At present, little is known about the exact impact of large-scale use of NPs, such as foliar use in agricultural fields, especially about their accumulation in the soil and toxicity to the ecosystem. Hence, the controlled and precise use of NPs should be a priority. In this sense, nano-priming is a highly controlled method compared to the foliar application of NPs. In other words, with nano-priming, it is possible to minimize environmental exposure to NPs, and nano-ecotoxicity.

## 8. Conclusions and Future Perspectives

Nano-priming could be a promising tool to mount desired physiological responses and productivity attributes in crops. The utility of nano-priming has been proven to increase seed vigor, germination, photosynthetic output, biomass, early growth, and yield in various valuable crops. Studies have shown that salinity tolerance and disease resistance responses can be successfully mounted in plants by nano-priming. Findings about the use of nano-priming methods in modulating antioxidant defenses and hormonal networks of plants are encouraging, and they can have several applications in plant-applied biotechnology. However, it is noteworthy that the physiological outcomes of various nano-priming techniques in plants are found to be inconsistent. They change according to the nanomaterials, their application concentrations, and plant species and varieties. Hence, it is crucial to optimize and validate the physiological outcome of each nano-priming method for each plant genotype, from lab to field.

Thus far, very few studies have been undertaken that are related to the uptake, transport, distribution, and accumulation of NPs in the nano-priming method. Moreover, studies related to the mechanism of action of NPs in seeds and plants related to seed germination, seedling, and plant growth, including their role in disease resistance and tolerance, are also scanty. Generating comprehensive knowledge about the above scientific gaps will help accept nano-priming methods unanimously as a potential tool for crop improvement. Phytotoxicity and environmental safety are two main concerns about the use of nanotechnology, including nano-priming in agriculture. These issues can be addressed by promoting the use of green nanomaterials and generating sound scientific knowledge about the interactions between engineered nanomaterials and plants in the context of their toxicity.

## Figures and Tables

**Table 1:** Impact of seed priming with various nanomaterials on physiological responses and productivity attributes of plants.

Sr. No.	Nanomaterials	Average Size of Nanomaterials (nm)	Seeds	Physiological/Productivity-Linked Response(s)	Effective Seed Priming Concentration and Time	Ref.
1.	Mn_3_O_4_ NPs	20	Corn	Increased germination, vigor, dry biomass, and length	20 mg L^−1^ (h)	[19]
2.	ZnO NPs	16.49	*Moringa oleifera* L.	Increased early growth and bioactive compounds	10 mg L^−1^ (1 h)	[20]
3.	ZnO NPs	100	Maize	Improved seed vigor index, germinationpercentage, shoot and root length, and fresh biomass	200 mg L^−1^ (24 h)	[21]
4.	ZnO NPs combined with sodium selenite and sodium selenate	<10	Direct-seeded rice	Enhanced seed vigor, metabolic profiles, nutrient uptake, growth, and yield	10 µmoL (24 h)	[22]
5.	FeO NPs	20–50	Rice	Improved seed germination and growth	20 mg L^−1^ (24 h)	[23]
6.	Ag NPs	36.5–171.3	Watermelon	Increased seed germination, growth, and yield	31.3 mg L^−1^ (12 h)	[11]
7.	Au NPs	30–113	Onion	Enhanced germination, growth, and yield	5.4 mg L^−1^ (12 h)	[24]
8.	Fe_2_O_3_ NPs	8–10	Chickpea	Increased seedling growth	<12 μg mL^−1^ (4–5 min.)	[25]
9.	Ag NPs	19.9–36.9	Cabbage	Accelerated seed germination speed, seedling development, yield, and nutritional quality	20 and 40 mg L^−1^ (15 h)	[26]
10.	Fe_2_O_3_ NPs	12–50	*Kobresia capillifolia* (Decne.) C.B.Clarke	Increased rubisco activity and photosynthetic rate	10–100 mg L^−1^ (12 h)	[27]
11.	Mn_2_O_3_ NPs	22–39	Watermelon	Modulated chlorophyll and antioxidant profiles	20 mg L^−1^ (14 h)	[15]
12.	Fe_2_O_3_ NPs	19–30	Watermelon	Modified the jasmonic acid and 12-oxo phytodienoic acid levels	20–160 mg L^−1^ (14 h)	[12]
13.	Polyacrylic acid-coated CeO_2_ NPs	9.2	Rapeseed	Increased salicylic acid level and ROS scavenging ability	0.1 mM (8 h)	[28]
14.	Chitosan nanoparticles containing Cu	174.2	Maize	Promoted early growth and enzymatic antioxidant defense	0.0625 mmol L^−1^	[29]
15.	Nanoscale zerovalent Fe	33.8	Rice	Regulated intracellular ROS levels, increased activity of hydrolytic, dehydrogenase, and antioxidant enzymes	20 mg L^−1^ (30 min.)	[30]
16.	Ag NPs	150	Rice	Enhanced hydrogen peroxide generation and antioxidant enzymes	10 mg L^−1^ (24 h)	[31]
17.	1,2,4-triazolyldithiocarbamate conjugated Ag NPs	45.48	Rice	Showed activity against *Fusarium fujikuroi*	100 mg L^−1^ (8 h)	[32]
18.	Se NPs	60.48–123.16	Tomato	Elicited resistance against tomato late blight disease	100 mg L^−1^ (4 h)	[33]
19.	SiO_2_ NPs	5–15	*Beta vulgaris* L. (beetroot)	Control *Meloidogyne incognita*, *Pectobacterium betavasculorum*, and *Rhizoctonia solani* disease complex of beetroot	200 mg L^−1^ (12 h)	[34]
20.	Cu–chitosan NPs	19–21	Wheat	Mitigated hyperosmotic stress and salinity	0.12% and 0.16% (8 h)	[35]
21.	Se and ZnO NPs	10–55 and ~20	*Brassica napus* L. (rapeseed)	Modulated the expression of ABA and GA genes during the germination stage; induced salinity tolerance by reducing the oxidative damage	150 µmol L^−1^ of Se-NPs and 100 mg L^−1^ of ZnO-NPs (8 h)	[36]
22.	Ag NPs	50–100	*Pennisetum glaucum* L. (pearl millet)	Enhanced salinity tolerance by reducing oxidative damage, reducing Na^+^ uptake, and maintaining the Na^+^/K^+^ ratio	20 mM (20 h)	[37]
23.	Chitosan nanoparticle	-	*Silybum marianum* (L.) Gaertn. (milk thistle)	Enhanced salt stress by increasing photosynthetic pigment synthesis, antioxidant enzyme activity, and free proline content	0.25% (6 h)	[38]
24.	Fe_2_O_3_ NPs	80	Wheat	Biofortified iron	25 mg L^−1^ (12 h)	[39]
25.	Si NPs and *Pseudomonas putida*	20–30	*Melissa officinalis* L.	Increased primary and secondary metabolites	100 mg L^−1^ (24 h)	[40]
26	ZnO NPs	30	Rice	Promoted early growth and resilience against cadmium toxicity	50 and 100 mg L^−1^ (20 h)	[41]
27.	ZnO NPs	20	Maize	Alleviated cobalt’s toxic effect by decreasing its uptake and improved photosynthetic apparatus	500 mg L^−1^ (24 h)	[42]
28.	Si NPs	-	Wheat	Increased growth chlorophyll contents, activity of enzymatic antioxidants, diminished oxidative stress, and reduced Cd contents	1200 mg L^−1^ (20 h)	[43]

## Data Availability

Not applicable.
